# Immunological Links to Nonspecific Effects of DTwP and BCG Vaccines on Infant Mortality

**DOI:** 10.1155/2011/706304

**Published:** 2011-05-05

**Authors:** Mogens Helweg Claesson

**Affiliations:** Laboratory for Experimental and Cellular Immunology, Department of International Health, Immunology and Microbiology, The Panum Institute, University of Copenhagen, Blegdamsvej 3, 2200 Copenhagen, Denmark

## Abstract

A number of mainly observational studies suggest that many African females below the age of one year die each year from the nonspecific effects of vaccination with diphtheria-tetanus toxoids and killed (whole-cell) *Bordetella pertussis* (DTwP). In contrast, similar studies suggest that many African females and males may have their lives saved each year by the nonspecific immunological benefits of Bacillus Calmette-Guerin (BCG) vaccination. From an immunological point of view, we hypothesise that the adverse effects of DTwP vaccine may occur because of the Th2-polarising effect of the aluminium phosphate adjuvant in the vaccine and because intramuscular administration of the vaccine may cause chronic inflammation at the site of injection. However, the Th1-polarising effect of BCG is likely to be beneficial. Sexual dimorphism affecting immune functions and vitamin A supplementation may influence both the deleterious and beneficial nonspecific effects of immunisation.

## 1. Background

The paper discusses the immunology behind the reported nonspecific effects of DTwP and BCG vaccination: increased childhood mortality rates after DTwP and decreased mortality rates after BCG vaccination given during the course of the WHO recommended vaccine programs implemented in Guinea-Bissau and other low-income countries [1, 2]. Both the detrimental and the beneficial effects of vaccination are strongest in females [3–7]. We speculate here that vaccine-induced changes in innate and adaptive immunity may play a role.

## 2. DTwP Vaccine

According to the WHO recommendations children in low-income countries are vaccinated against diphtheria, tetanus, and pertussis at 6, 10, and 14 weeks of age. Most countries use a whole-cell *Bordetella pertussis* vaccine. A typical DTwP vaccine dose (0.5 mL) from the Serum Institute of India contains diphtheria toxoid (25 Lf), tetanus toxoid (5 Lf), and *pertussis* toxoid (4 IU), aluminium phosphate (1.5 mg), and a preservative, thiomersal (0.01%). Aluminium phosphate acts primarily as an antigen-adsorbing and Th2-polarizing adjuvant [8]. Intramuscular vaccine injection results in a palpable sore induration at the site of injection in 70% of children. In 9% of the children the induration lasts 4 weeks and there is a significant correlation between local reactivity and fever (J. Agergaard, personal communication). 

## 3. Increased Mortality after DTwP Vaccination

The major causes of death in general among children in Guinea-Bissau are related to gastrointestinal infections with diarrhoea and dehydration, pneumonia and septicaemia, whereas the risk of death from pertussis, even in unimmunized high-mortality areas, is relatively low [2]. Up to recently, malaria has also been a major cause of death in Guinea-Bissau (C. Stabell Benn, personal communication). 

A number of observational studies in Guinea-Bissau have shown that DTwP vaccination is associated with an increased mortality in female infants [3–5, 9] most probably reflecting higher rates of fatal diarrhoea and pneumonia among vaccinated children. In line with this, observational studies on the morbidity of vaccinated versus nonvaccinated children have shown that DTwP vaccination is associated with increased incidence of colonisation and infections with endemically occurring rotavirus [10] and *Cryptosporidium parvum* [11] in females. Both microorganisms cause extensive diarrhoea, dehydration, and death. Furthermore, recently published randomised trials found increased risks of growth retardation, *Chlamydia pneumoniae *infections, and morbidity (diarrhoea) in girls who had received a DTwP booster vaccine [12, 13]. It should be noted that *Chlamydia *infection* per* *se* is unlikely to cause the high proportions of fatal pneumonia in infant females after DTwP vaccination as observed by Veirum et al. [9].

## 4. Immunology

Are there immunological mechanisms to explain the deleterious nonspecific effects of intramuscular DTwP vaccination? First it should be stressed that the DTwP vaccine in use provides efficient protection against the three diseases targeted by the vaccine. However, the intramuscular injection, which leads to a Th2-dominated protective antibody response [8], might be the problem *per se*. Thus, the intramuscular vaccine deposit, which slowly releases the aluminium-adsorbed vaccine antigens, causes long-lasting local induration at the site of vaccination in some children. The induration probably reflects a local inflammatory reaction with accumulation of phagocytic and lymphoid cells including bone marrow-derived macrophages. These cells are triggered and activated by vaccine antigens to secrete inflammatory and fever-inducing cytokines like TNF*α*, IL-6, and IL1*β* [14]*.* Elevated circulating levels of these cytokines may be highly correlated with mortality in infants with severe endemic infections (see above) [15]. Ongoing inflammation might also lead to macrophage reprogramming toward a type 2 macrophage profile secreting immunosuppressive cytokines such as IL-10 and TGF*β* [15]. Such reprogramming would, in concert with the Th2-polarizing effect of aluminium phosphate [8], dampen protective cellular immunity in general. Enhanced systemic levels of IL-10 and TGF*β* together with insufficient cellular immunity may lead to an increased susceptibility to the endemic pathogens mentioned above. 

Heterogenous cell populations of myeloid-derived suppressor cells (MDSCs) are primarily known for their immunosuppressive and tumor-promoting effects within and outside a tumor environment [16]. However, MDSC-like cells may also be recruited and respond to stress and danger signals including DTwP vaccine-induced inflammation (overview in [17]), and they may accumulate in and disseminate from the inflammatory site of the DTwP injection. Like type 2 macrophages, MDSC-like cells secrete immunosuppressive cytokines such as TGF*β* and IL-10 which are involved in recruitment of regulatory T cells (Treg). Treg activity may decrease levels of protective immunity thereby increasing the risk of morbidity and mortality due to endemic pathogens [10, 11, 18, 19]. In addition, pertussis toxin (PT), present in DTwP, changes levels of Th1/Th2 cytokines [20] and might decrease resistance to infectious pathogens. Thus, animals primed with purified and detoxified PT or DTwP followed by exposure to respiratory syncytial virus showed an increased morbidity (weight loss) and mortality compared with mice not primed with PT prior to virus challenge [21].

## 5. Nonspecific Decreased Mortality after BCG Vaccination

Protection by one invading microorganism, for example, by vaccination, against another unrelated microorganism is recognized as heterologous immunity (HI) [22]. HI also implicates that the pool of specific immunological memory of an individual will be changed for each new infection or vaccine experienced by the individual. 

BCG vaccination may lead to HI since it appears to have some positive survival effects not related to its specific protection against tuberculosis. Thus, the reduced overall mortality from BCG vaccinations, not related to TB, has been estimated to be 25% [1]. Likewise, BCG vaccination in children less than two years old in low-income countries appears to provide significant protection against infectious diseases with a deadly outcome [6, 7]. This benefit appears to be strongest for females [6, 7]. In a randomised trial among low-birth-weight children who do not normally receive BCG at birth, BCG administered at birth was associated with a 45% (11–65%) survival benefit in the first month of life (P. Aaby et al. submitted for publication). 

## 6. Immunology

BCG vaccination in young infants nonspecifically increases polio-specific antibody responses after polio vaccination but not after diphtheria or tetanus vaccination [23]. This nonspecific, immune-enhancing effect of BCG is probably due to stimulation of innate immunity, that is, enhanced antigen presenting activity and efficacy. The mycobacterial cell wall activates and differentiates dendritic cells and macrophages through TLR2, -4, and -9 ligation increasing their functional activity [23–26] and polarizing towards Th1-type immune responses with increased levels of IFN*γ*, TNF*α*, and other inflammatory cytokines. This may in turn enhance immune responses towards infections other than tuberculosis. In addition, the TLR2 and -4 agonist activities of BCG lead to activation of inducible nitric oxide synthase (iNOS) in macrophages triggering their capacity to kill intracellular microorganisms. These changes may lead to the more general protection against endemic infectious diseases and thereby explain the decreased mortality rates among BCG-vaccinated children.

## 7. Cutaneous *versus* Intramuscular Administration and Aluminium Adjuvant

Subcutaneous and intradermally injected vaccines against BCG, measles, and smallpox are taken up and processed locally by macrophages and dendritic cells at the site of injection. The cells reach the regional lymph nodes via afferent lymphatics and thus the physiologically correct microenvironment for priming of T and B cells. Heterologous immunity after vaccine-induced activation of regional lymph nodes might well lead to an increased overall “immune awareness” at the systemic level. A “natural” nonspecific effect of cutaneous vaccinations with live microorganisms might explain the overall positive effect on the mortality rate not only following BCG vaccination but also following vaccinations against smallpox [27, 28] and measles [29, 30]. 

On the contrary, intramuscular injection of DTwP aluminium adsorbed antigens might stick to the site of injection and here recruit inflammatory cells including antigen-processing cells. Antigen-loaded cells will leave the inflammatory site by the lymph as well as by the rich capillary bed of skeletal muscles and via this latter route reach the spleen, an organ in which robust antibody responses specific for the vaccine antigens are generated. The Th2-polarizing effect of aluminium phosphate together with recruitment of myeloid-derived suppressor cells might at the same time lower the overall efficacy of protective cellular immunity necessary for elimination of intracellular, potentially lethal infectious microorganisms in the environment, as discussed above.

## 8. Sex Differences in Nonspecific Vaccine Effects

As mentioned above, the mortality after vaccination is markedly different in females and males: DTwP vaccination increases overall female but not male mortality [3–5], whereas the heterologous effects of BCG and measles vaccines reduce females mortality with less significant effects in males [6, 7, 29, 30]. Immune dimorphism, that is, the differences in immune responses and regulation between sexes, is primarily thought to be caused by the immune modulatory effects of the sex hormones [31, 32]. In young male and female infants the different levels of sex hormones may result in both quantitative and qualitative differences in immunity between the two sexes. In 0-to-2-year old females, levels of plasma estradiol are comparable to levels of the late puberty or adult mid-follicular phase [33, 34], whilst in 0–6-month-old males, the plasma testosterone levels approach those of adults [34]. In particular, estradiol levels are high in premature girls [34]. The Th1/Th2 cytokine balance is greatly influenced by sex hormones. Th1 cells secrete proinflammatory cytokines like IFN*γ* and TNF*α*, whereas Th2 cells secrete anti-inflammatory cytokines like IL-4, IL-5, IL-10, and IL-13. Importantly, the two Th subsets are mutually inhibitory. Physiological levels of estradiol stimulate in particular humoral immune responses, whereas testosterone inhibits such responses [31, 32, 35]. The effects of estradiol on the generation of Th2-derived anti-inflammatory cytokines in infant females might therefore inhibit secretion of Th1-derived inflammatory cytokines and skew the immune response towards Th2-type responses including Treg stimulation (for overview, see [36] and [37]). Upon DTwP vaccination of baby females, as compared to males, the adjuvant-containing vaccine *per se* (as discussed above) alongside the estradiol-rich environment may lead to a more strongly polarized Th2 immunity at the expense of nonspecific and protective Th1-type immunity. In contrast, BCG vaccination, which by itself enhances the Th1 arm, as discussed above, might outweigh the negative effects of estradiol on Th1-type responses in female infants but leave their Th2-type responses intact. Thus, the BCG vaccinated females will gain an overall broader general immune protection than the males who lack the strong Th2-polarizing effect of estradiol.

## 9. Vitamin A Supplementation

In spite of numerous studies, there is still only weak evidence for any effect of micronutrient supplementation on vaccine efficacy (reviewed in [38]). However, recent studies strongly suggest that supplementation with vitamin A, iron, and zinc enhances both the detrimental nonspecific effects of DTwP vaccination in infant girls and the beneficial effects of BCG vaccination [39, 40]. Vitamin A supplementation has been shown to enhance the specific antibody responses in infants vaccinated against diphtheria [41] and tetanus [42]. Hypothetically, this specific antibody-enhancing effect of vitamin A might outcompete protective responses to endemic pathogens (see above), thus explaining the harmful effect of vitamin A supplementation in DTwP vaccines. Vitamin A supplementation might also increase the activity of regulatory T cells [43] and thereby potentially downmodulate protective adaptive responses against endemic pathogens. Vitamin A supplementation has also been shown to increase NK-cell activity in immunosuppressed infants [44], which together with the nonspecific stimulatory effect of BCG on the innate immune system [24–26] might improve the beneficial survival effect observed after BCG vaccination.

## 10. Conclusions and Suggestions

 The deleterious effect of DTwP vaccination in infant females and the beneficial outcomes of BCG vaccination might reflect nonspecific immune-polarizing and nonintended regulatory effects of vaccines and sex hormones in early childhood. The data from West Africa on the nonspecific effects of DTwP and BCG vaccination in young children discussed here suggest (1) replacement of intramuscular DTwP vaccination with cutaneous vaccination or development of a DTwP vaccine live bacterial carrier such as BCG [45] which may reduce nonspecific negative effects, (2) that BCG vaccination should be performed within the first week after birth to maximally improve its nonspecific effect on overall survival, (3) that many new vaccines against tuberculosis under development should be tested not only for their overall effects on tuberculosis but also for their overall effect on infant mortality, (4) that research into sex differences of immune functions in infant males and females is essential when developing new vaccines; (5) that research into immunological mechanisms is of major importance to understanding the interaction between nutrient supplementation and vaccine efficacy. 

In low-income countries with high rates of childhood mortality, the nonspecific effects of routine vaccinations are likely to be of increased importance due to the high disease burden. The nonspecific vaccine effects should therefore be a high-priority research area and be considered in the future planning of immunization programs in these countries to enhance the beneficial impact of immunizations on child survival.

##  Conflict of Interests 

The author has no relevant affiliations or financial involvement with any organization of entity with financial interest in or financial conflict with the subject matter discussed in this paper. No writing assistance was utilized in the production of this paper.
